# Holocord syringomyelia associated with von Hippel-Lindau disease: a case series

**DOI:** 10.3389/fsurg.2026.1896269

**Published:** 2026-07-09

**Authors:** Shijie Li, He Wang, Tao Liu, Xiangdong Yin, Shengli Shen, Liang Li, Hongzhou Duan

**Affiliations:** 1 Department of Neurosurgery, Peking University First Hospital, Beijing, China; 2 Department of Radiology, Peking University First Hospital, Beijing, China

**Keywords:** hemangioblastoma, spinal cord, holocord syringomyelia, treatment, von Hippel–Lindau disease

## Abstract

**Introduction:**

Holocord syringomyelia is a rare and severe form of syringomyelia, often associated with disruptions in cerebrospinal fluid (CSF) dynamics. In patients with von Hippel-Lindau (VHL) disease, multifocal spinal hemangioblastomas may contribute to the development of extensive syringomyelia. However, the pathogenesis and optimal management of VHL-associated holocord syringomyelia remain poorly defined.

**Materials and methods:**

A retrospective review was conducted on 136 patients diagnosed with VHL disease at our institution. Four patients with VHL-associated holocord syringomyelia were identified. Their clinical presentations, imaging characteristics, treatment strategies, and outcomes were systematically analyzed.

**Results:**

All four patients exhibited holocord syringomyelia accompanied by multiple intramedullary hemangioblastomas. Clinical manifestations varied markedly, ranging from completely asymptomatic to progressive neurological deficits. Surgical resection of selected symptomatic lesions resulted in clinical improvement in some patients, but radiological resolution of the syrinx was inconsistent and often incomplete.

**Conclusion:**

Holocord syringomyelia in VHL disease likely results from the cumulative effects of multiple segmental lesions rather than a single causative tumor. Management should prioritize symptom control and individualized decision-making, rather than aggressive treatment of the syrinx itself.

## 1 Introduction

Syringomyelia is a chronic and progressive spinal cord disorder, characterized by the formation of fluid-filled cavities within the spinal cord parenchyma. These cavities, filled with cerebrospinal fluid-like fluid, may gradually expand and lead to spinal cord dysfunction, consequently resulting in diverse neurological deficits. Although syrinx formation can occur at any spinal level, holocord syringomyelia is a rare yet severe manifestation of the disease, in which the cavity extends longitudinally from the upper cervical cord to the conus medullaris.

Von Hippel-Lindau (VHL) disease is a rare autosomal dominant hereditary disorder, characterized by the development of benign and malignant tumors in multiple organ systems. Spinal hemangioblastoma is a common manifestation of VHL disease, with an incidence of around one-half (1, 2). These spinal hemangioblastomas are often multifocal and can involve multiple spinal segments. By compressing or obstructing CSF pathways, they are frequently associated with focal syringomyelia and may give rise to a series of neurological symptoms. Holocord syringomyelia is an exceptionally rare presentation in patients with VHL-associated spinal hemangioblastomas and has been only sporadically reported in the literature. In this study, we will present four rare cases of VHL-associated holocord syringomyelia, and review the relevant literature to further elucidate the possible underlying pathophysiological mechanisms and optimize management strategies for this rare but clinically significant condition.

## 2 Case series

We retrospectively reviewed 136 patients diagnosed with VHL disease at our institution, among whom four cases (2.9%) were found to have holocord syringomyelia. In all four patients, spinal magnetic resonance imaging (MRI) revealed a holocord syrinx accompanied by multiple intramedullary hemangioblastomas. The diagnosis of VHL disease was based on family history, clinical manifestations, and confirmatory genetic testing. The four patients differed in terms of clinical management strategies and outcomes. Their clinical features, treatment approaches, and follow-up results are reported as follows.

### 2.1 Case 1

**Figure 1:**
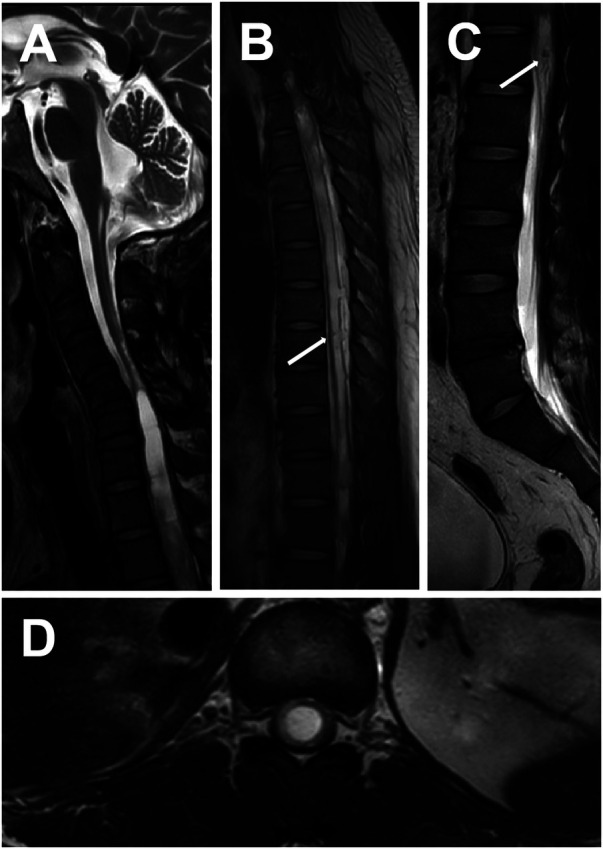
Spinal MRI of Case 1 demonstrating a holocord syrinx involving the entire spinal cord **(A–C)**. Intramedullary hemangioblastomas are indicated by white arrows in **(B,C)**. Axial MRI at the T12–L1 level demonstrating the maximal diameter of the syrinx **(D)**.

A 42-year-old male with a documented history of multiple VHL-associated tumors presented to the ophthalmology clinic of our institution, reporting blurred vision in the right eye. He had previously undergone surgical resection of a left cerebellar hemangioblastoma and was confirmed of VHL disease at another hospital. A systemic assessment including non-contrast spine MRI was conducted. The imaging examination findings indicated dilation of the central canal from the lower medulla to the C5 level, along with a huge syrinx extending from C6 to the conus medullaris, featuring internal septations (Figures 1A–D). Moreover, multiple intramedullary nodular lesions were detected at the T4, T6, T9, and L1 levels, which were suggestive of spinal hemangioblastomas; however, due to the patient's poor renal function, contrast-enhanced MRI was not carried out. Despite the presence of an extensive holocord syrinx, the patient did not manifest any neurological deficits related to spinal cord involvement. Therefore, close clinical follow-up was implemented. The patient's symptoms have remained essentially unchanged throughout the two-year telephone and outpatient follow-up period.

### 2.2 Case 2

**Figure 2:**
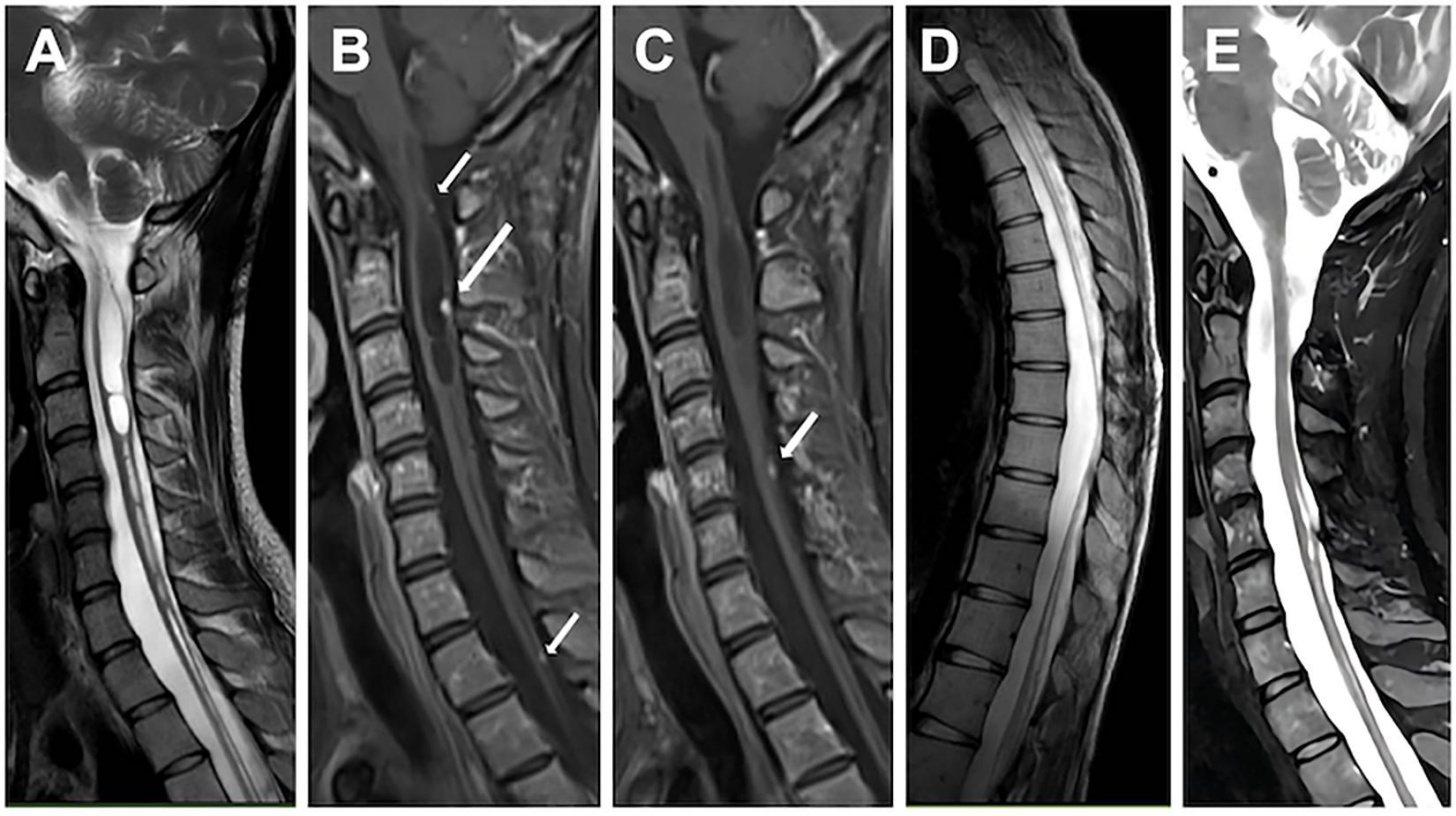
Spinal MRI of Case 2 demonstrating holocord syrinx with multiple intramedullary hemangioblastomas on preoperative imaging **(A–D)**, with contrast-enhanced sequences shown in **(B,C)**. White arrows indicate the distribution of the lesions. Follow-up MRI at 5 years postoperatively showing the evolution of the cervical syrinx **(E)**.

The second case was a 23-year-old female, she was previously diagnosed of VHL disease based on multiple VHL-associated tumors, a documented family history and a positive gene examination. The patient had previously undergone two times resection of right cerebellar hemangioblastoma and one time resection of a T7–T8 spinal hemangioblastoma. Post-operatively, she continued to present with increased muscle tone in both lower extremities. During routine follow-up, a spine MRI examination revealed a holocord syrinx originating from the lower medulla, accompanied by multiple intramedullary hemangioblastomas at the C2–T1 levels (Figures 2A–D). Given that the patient's clinical symptoms might be attributed to the cervical intramedullary lesions, the patient was admitted, and a surgical resection of the cervical tumors was performed.

A midline posterior approach from C1 to C5 was implemented. After the removal of the posterior arch of C1 and the exposure of the C2 to C5 laminae, the dura was opened. A reddish, well-demarcated nodular lesion with a diameter of approximately 6 mm was identified on the right dorsal spinal cord at the C2 level. The lesion was firm, highly vascular, and adherent to the surrounding spinal cord, with prominent feeding arteries and draining veins. Carefully microsurgical dissection was performed, and the feeding arteries and draining veins were sequentially coagulated and divided, enabling *en bloc* resection of the lesion. Following tumor removal, the syrinx cavity was shrinked, and a significant collapse of the spinal cord was observed. Using the same approach, a further exploration of the C1 and C5 segments facilitated the removal of four additional nodular lesions. The dura was closed in a watertight manner, the laminae were repositioned, and the wound was closed in layers. Two months post-operatively, the patient showed significant improvement in lower limb motor function. Follow-up imaging at 6 months revealed a marked reduction of the syrinx above the C4 level (Figure 2E).

Although intracranial hemangioblastomas and extracranial manifestations of VHL disease continued to progress, the patient underwent an additional resection of a left cerebellar hemangioblastoma 3 years later due to dizziness and nausea. Notably, during follow-up, there was no further deterioration in spinal cord–related neurological function, and imaging demonstrated sustained stability of the syrinx.

### 2.3 Case 3

**Figure 3:**
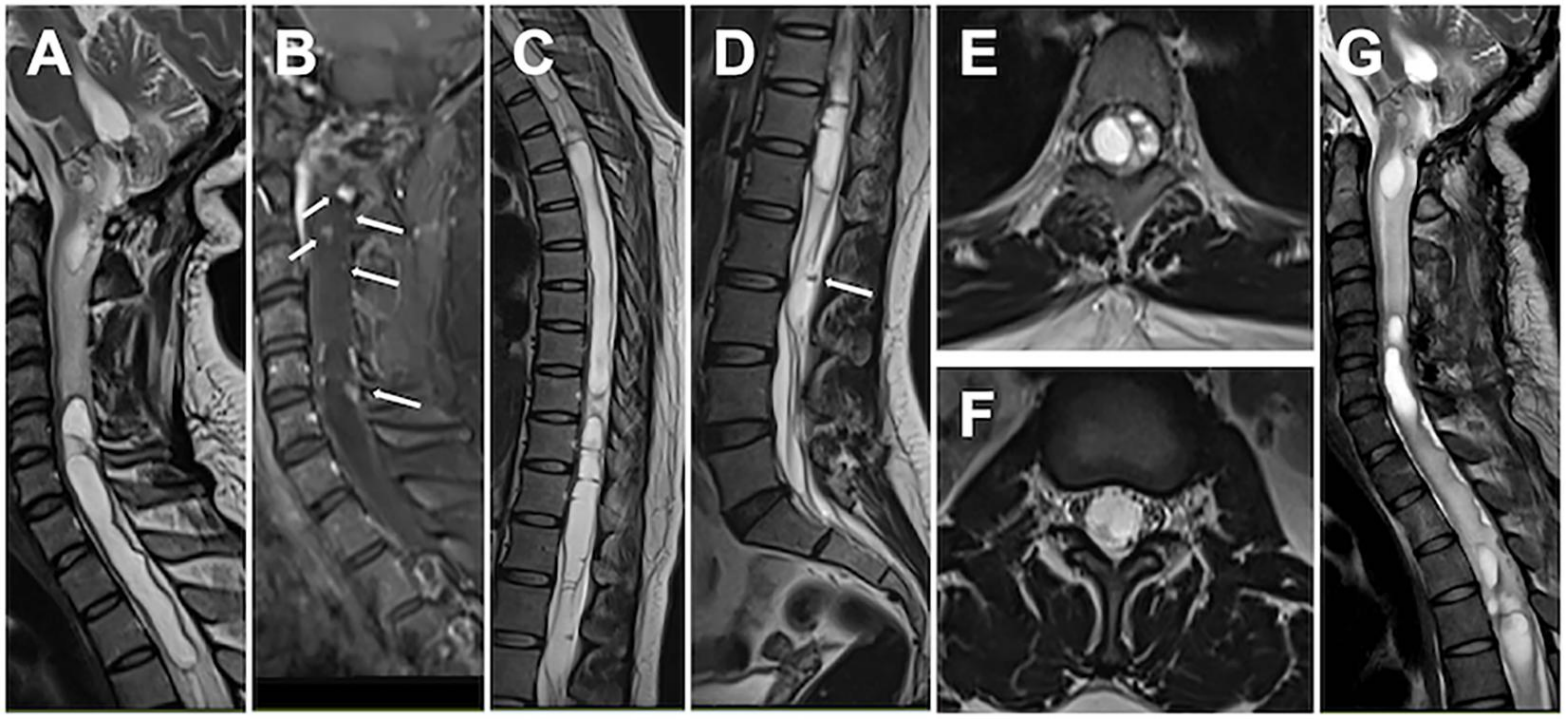
Spinal MRI of Case 3 demonstrating holocord syrinx from medulla oblongata to cone **(A–D)**, with multiple contrast-enhanced intramedullary hemangioblastomas (white arrow). Axial MRI at the T4 and L2–3 levels showing the syrinx cavity in association with focal intramedullary lesions **(E,F)**. Follow-up cervical MRI demonstrating no resolution of the syrinx at the surgically treated level **(G)**.

A 33-year-old woman with a prior diagnosis of VHL disease presented to our institution with progressively worsening neck and shoulder pain. Cerebral and a whole-spine MRI examination revealed an extensive holocord syrinx involving nearly the entire spinal cord, extending from C2 level and down to L3 level (Figures 3A–F). In addition, multiple hemangioblastomas were identified in the cerebellar vermis, bilateral cerebellar hemispheres, dorsal medulla, and spinal cord. Intramedullary spinal lesions were distributed at the C2, C5–C6, T1–T4, and L2 levels.

Given that the patient's predominant symptoms were most likely attributable to focal cervical C5-6 hemangioblastoma, a posterior approach was performed at the C5–C6 level with laminectomy and durotomy, followed by gross total resection of four intramedullary tumors. A fenestration of spinal cord was then performed at the most thinned dorsal spinal cord near the dorsal root entry zone. Upon release of clear cerebrospinal fluid, the spinal cord collapsed markedly. Postoperatively, the patient experienced improvement in neck and shoulder pain. However, 2 years' follow-up cervical MRI demonstrated no significant change in the extent or morphology of the syrinx compared with preoperative imaging (Figure 3G).

### 2.4 Case 4

**Figure 4:**
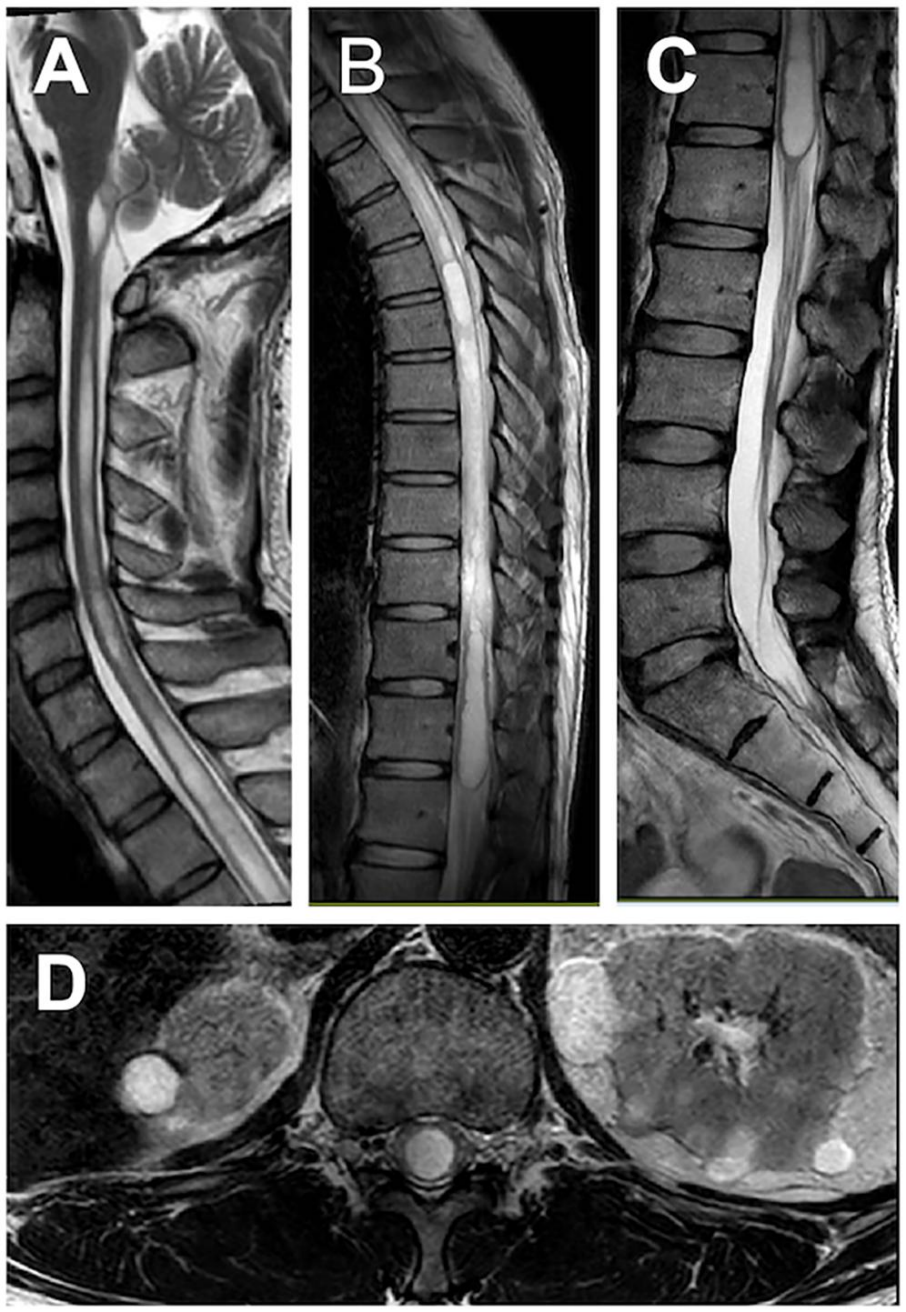
Spinal MRI of Case 4 demonstrating a holocord syrinx involving the entire spinal cord **(A–C)**. Axial MRI at the T12–L1 level showing the maximal diameter of the syrinx **(D)**.

A 43-year-old male was clinically diagnosed with VHL disease based on the presence of multisystem tumors and confirmed by gene examination. Follow-up spinal MRI examination demonstrated a holocord syrinx spanning from the medulla oblongata to the conus medullaris (T12) (Figures 4A–D), manifested as a continuous cavity accompanied by segmental cystic dilatations. Non-contrast MRI indicated multiple intramedullary lesions located at the dorsal medulla oblongata, as well as at the T2 and T5 levels, which were suspected as the spinal hemangioblastomas. Despite the extensive radiological involvement of the entire spinal cord, the patient maintained neurological integrity, without any motor, sensory, or autonomic dysfunctions. Therefore, no surgical intervention was performed, and only close observation and follow-up were carried out. No significant symptom changes noted during 2-year outpatient follow-up.

## 3 Discussion

Syringomyelia is an infrequent disorder characterized by the development of abnormal fluid-filled cavities within the spinal cord. Common causes encompass congenital anomalies such as Chiari malformation and tethered cord syndrome (3), neoplastic lesions such as ependymomas and astrocytomas, as well as traumatic spinal cord injury (4).

At present, several theoretical models have been proposed to explain the pathogenesis of syringomyelia, which is closely related to the disturbance of cerebrospinal fluid (CSF) dynamics (5, 6) and the imbalance of intramedullary and extramedullary pressure gradients (7). Using phase-contrast MRI, our previous study revealed that altered CSF flow dynamics are closely associated with the development of syringomyelia (8), providing a mechanistic basis for understanding holocord syrinx formation in VHL disease.

Holocord syringomyelia is a special subtype of syringomyelia. Patients with this condition might frequently present with more severe clinical symptoms. However, there is no specific study on the etiology and pathogenesis of holocord syringomyelia.

Traditionally, the formation of syringomyelia associated with spinal hemangioblastomas has been attributed to tumor mass effectc (1). However, the pathogenesis of syrinx formation in VHL disease is likely more complex, involving the combined effects of multifocal lesions, chronic changes in CSF hydrodynamics, and an abnormal tumor microenvironment. VHL disease is an autosomal dominant hereditary disorder caused by mutations in the VHL gene located on chromosome 3p. The loss of function of the VHL protein leads to the constitutive activation of the hypoxia-inducible factor (HIF) pathway, leading to upregulation of downstream targets such as vascular endothelial growth factor (VEGF) and the development of highly vascularized tumors. Importantly, unlike spinal ependymomas, which may disseminate via CSF pathways, or schwannomas, which may exhibit positional mobility, VHL-associated hemangioblastomas are characterized by multifocal independent tumorigenesis rather than metastatic spread. This biological distinction has direct implications for surgical planning: resection of individual symptomatic lesions, rather than attempts to eradicate all detectable tumors, constitutes a rational therapeutic strategy. Pathological studies have indicated that the rich vascularity and increased vascular permeability of spinal hemangioblastomas facilitate extravasation of plasma components into the surrounding neural tissue. As fluid gradually accumulates, local edema and tissue compression lead to the development of peritumoral cystic cavities. These cysts can exert additional mass effect on the subarachnoid space, further impairing CSF circulation and promoting the influx of fluid into the spinal cord parenchyma (9, 10). When such fluid-filled cavities extend within the spinal cord, a syrinx is formed.

In addition, both the location and multiplicity of lesions influence the extent of syrinx formation. Clinical findings indicate that tumors located at higher spinal levels, especially in the cervical cord, are more likely to be associated with extensive syrinx involvement (11). Furthermore, the multifocal characteristic of VHL-associated lesions may promote the longitudinal extension and confluence of syrinx cavities, increasing the development of holocord syringomyelia (1, 12). Recurrent tumor-related hemorrhage and prior surgical interventions in VHL patients may also lead to subarachnoid adhesions, thereby disrupting CSF flow and further contributing to syrinx formation.

With syringomyelia, patients may exhibit dissociated sensory deficit, which is characterized by the impairment of pain and temperature perception below a specific segmental level, whereas light touch and proprioception are relatively retained (12). As the syrinx expands and exerts pressure on the lateral columns of the spinal cord, signs of upper motor neuron typically emerge, presenting as spastic paralysis, encompassing limb weakness, elevated muscle tone, hyperreflexia, and positive pathological reflexes (12–15). Involvement of the anterior horn may result in segmental lower motor neuron signs, such as muscle atrophy, weakness, and diminished reflexes (15, 16). When the syrinx extends rostrally into the medulla oblongata, symptoms related to brainstem and high cervical cord dysfunction may occur, including facial sensory deficits, lingual weakness, dysphagia, and, in severe cases, Horner syndrome or impairment of cardiorespiratory centers (1, 12). If the lesion involves the conus medullaris, patients may develop lower limb motor and sensory deficits in conjunction with bladder and bowel dysfunction (12–17). In VHL disease, hemangioblastomas frequently involve the cerebellum and brainstem; therefore, in addition to spinal cord–related manifestations, patients may also present with symptoms such as headache, dizziness, and ataxia (18). The clinical manifestations of VHL-associated holocord syringomyelia are highly heterogeneous and depend on both the extent of syrinx involvement and the location of the underlying lesions. In the present series, Case 2 exhibited features of spastic paraparesis, including lower limb dysfunction and increased muscle tone. Case 3 presented with neck and shoulder pain attributable to compression from multiple spinal lesions. In contrast, Cases 1 and 4 were clinically asymptomatic despite significant radiological involvement. However, due to substantial overlap in symptomatology, it is often difficult to delineate whether clinical deficits arise primarily from the syrinx itself or from the associated tumor. Fundamentally, both mechanisms converge on a common pathophysiological pathway—compression of the spinal cord leading to neurological impairment.

Incidental spinal hemangioblastomas are frequently encountered in VHL patients during routine surveillance imaging, as exemplified by Cases 1 and 4 in our series, who remained completely asymptomatic despite extensive holocord involvement. The management of these incidental lesions poses a clinical dilemma, as prophylactic resection of all detectable tumors is neither feasible nor justified given the multifocal nature of the disease and the potential for cumulative surgical morbidity. Based on our experience and a synthesis of the literature, we propose the following indications for surgical intervention in VHL-associated spinal hemangioblastomas: (1) presence of progressive neurological deficits attributable to a specific lesion (19, 20); (2) documented radiological growth of a tumor, particularly if it exceeds 3 cm in diameter or demonstrates accelerated expansion (21); (3) significant mass effect with cord compression or peritumoral edema; and (4) patient preference after thorough informed consent. Conversely, conservative management with regular clinical and MRI surveillance is recommended for incidental, asymptomatic, or stable lesions, regardless of their size or extent, provided no neurological compromise exists (6). This symptom- and growth-oriented strategy, rather than an indiscriminate approach to all detected lesions, helps minimize unnecessary surgical interventions while ensuring timely treatment when clinically indicated.

For syringomyelia secondary to spinal hemangioblastomas, an individualized treatment is recommended, integrating microsurgical tumor resection, radiotherapy, targeted therapy, and immunotherapy. Syrinx-subarachnoid shunting was not employed in our series, as we consider tumor-targeted resection the primary strategy, given the limited and controversial role of CSF diversion in VHL-associated multifocal disease. Radiotherapy for spinal hemangioblastomas remains debated. To date, no randomized controlled trial compared radiotherapy with surgical outcomes in patients with hemangioblastomas was performed. Nevertheless, more and more observational study data showed the function of stereotactic radiosurgery in selected patients, particularly those with multifocal lesions not amenable to surgical resection (22). Prophylactic radiotherapy is not recommended in asymptomatic patients (23). Belzutifan, the first systemic therapy approved for VHL disease, exerts its effect by inhibiting HIF-2*α* dimerization, thereby blocking aberrant activation of the HIF signaling pathway and downregulating pro-tumorigenic factors such as VEGF, erythropoietin, and glucose transporter 1 (24). The U.S. Food and Drug Administration (FDA) has approved belzutifan for the treatment of adult patients with VHL disease who have hemangioblastomas that do not require immediate surgical intervention. Anti-angiogenic agents, such as pazopanib and sunitinib, have exhibited limited efficacy in this setting and are not considered in first-line treatment options.

In patients with syringomyelia caused by sporadic spinal hemangioblastomas, the alteration in CSF hydrodynamic patterns is relatively simple. Gross total resection of the tumor eliminates the underlying cause and often results in syrinx resolution or collapse (25). In contrast, patients with VHL-associated multifocal spinal hemangioblastomas exhibit a more heterogeneous clinical course. As illustrated in Cases 1 and 4, neurological function may remain relatively stable during long-term follow-up despite extensive holocord involvement. Moreover, the impact of tumor-targeted surgical intervention on syrinx exhibits substantial individual variability. Despite both Case 2 and Case 3 demonstrating postoperative symptomatic amelioration, the syrinx size diminished in Case 2, but with no notable radiological alteration detected in Case 3.

To contextualize our findings within the existing literature, we summarized previously reported cases of holocord syringomyelia associated with spinal hemangioblastomas, including sporadic and VHL-related cases (Table 1). This comparison highlights several key observations. First, both sporadic and VHL-associated cases can present with extensive holocord involvement, but VHL patients more frequently harbor multifocal tumors. Second, surgical resection of the causative lesion often leads to functional improvement, yet radiological resolution of the syrinx is inconsistent—ranging from marked collapse to no significant change. Third, asymptomatic holocord syrinx, as seen in two of our VHL patients, is not rare and supports a conservative approach. Collectively, these data reinforce the notion that holocord syringomyelia in VHL disease results from chronic, multifactorial CSF dynamic disturbances, where numerous segmental syrinxes coalesce into a holocord cavity, rather than a single obstructive tumor.

**Table 1:** Summary of the clinical characteristics of holocord syringomyelia associated with spinal hemangioblastomas based on previously published reports and the present study.

Reference	Age(y)/Sex	Type	Tumor Location	Syrinx Distribution	Preoperative Presentation	Treatment	Postoperative Functional Outcome	Postoperative Syrinx Changes
Knoop et al., 2019 (18)	37/M	VHL	Cerebellum， T1, T2, T11/12 junction	Cervicomedullary junction–L1	–	Staged surgery (1st: GTR + fenestration; 2nd: shunt; 3rd: GTR + RT)	Deteriorated	Decreased
Mortazavi et al., 2021 (1)	30/M	VHL	Medulla， C3–4， T10	C1–4, C6–conus medullaris	SA, BS	Multiple tumor resection	Markedly improved	Decreased
Borkar et al., 2009 (13)	38/F	Sporadic	C7–T1	Medulla–L1	SQP, UBD	Gross total resection	Improved	Not reported
Munjal, 2018 (14)	40/M	Sporadic	T12–L1	C2–T11	SA, SPP, UBD	Gross total resection	Stable	Not reported
Dutta et al., 2018 (12)	21/F	Sporadic	T4	Cervicomedullary junction–T12	DSL, SQP, BS, UBD	Near-total resection	Improved	Not reported
Cosgrove et al., 2015 (26)	50/M	Sporadic	T6–T7	Lower medulla – conus medullaris	SA, SPP	Not reported	Not reported	Not reported
Chu et al., 2023 (16)	29/F	Sporadic	L1	C2–L1	SA	Conservative treatment	Not reported	Not reported
Pai & Krishna, 2003 (15)	17/M	Sporadic	Conus medullaris	C2–conus medullaris	FQP, UBD	Gross total resection	Markedly improved	Resolved
	35/M	Sporadic	T8	Holocord syrinx	SA, SP	Gross total resection	Improved	Not reported
Bansal et al., 2023 (17)	32/F	Sporadic	T9	C2–T12	LSA/LSW	Gross total resection	Improved	Decreased
	21/M	Sporadic	T6–T7	Cervicomedullary junction–T11	SA, SPP, UBD	Gross total resection	Improved	Resolved
Our study	42/M	VHL	T4, T6, T9, L1	Cervicomedullary junction–C5, C6–L1	–	Regular follow-up	Stable	None
	23/F	VHL	C2–T1	Cervicomedullary junction–L1	SPP	Multiple tumor resection + fenestration	Improved	Decreased
	33/F	VHL	C2, C5–6, T1–4	C2, C4–L3	SA	Multiple tumor resection + fenestration	Improved	Stable
	43/M	VHL	Medulla, T2, T5	Medulla–T12	–	Regular follow-up	Stable	None

M, male; F, Female; DSL, dissociated sensory loss; VHL, Von Hippel-Lindau Disease; UBD, urinary/bowel dysfunction; SA, sensory abnormality; LSA, left sensory abnormality; LSW, left side weakness; FQP, flaccid quadriplegia; SQP, spastic quadriplegia; SPP, spastic paraplegia; SP, spastic paralysis; BS, brainstem symptoms.

The novelty of our study is twofold. First, we propose that holocord syringomyelia in VHL disease results from cumulative effects of multiple segmental hemangioblastomas coalescing into a continuous cavity—a mechanism not previously emphasized. Second, our observations demonstrate that clinical status is driven by tumor burden and location, not syrinx morphology, supporting a symptom-oriented strategy that targets symptomatic tumors rather than the syrinx itself. This individualized approach, based on our case series, has not been systematically addressed before.

There are many limitations in our study. First of all, the condition of holocord syringomyelia examined in this study is rare, and the number of cases included is limited. In addition, the retrospective nature of this study and incomplete initial imaging records from outside institutions prevented us from determining the chronological order of cerebellar vs. spinal lesion manifestation, which would require prospective longitudinal surveillance from the time of genetic diagnosis. Secondly, the duration of follow-up varied among patients and did not fully conform to recommended surveillance protocols for VHL disease. In addition, long-term, continuous MRI follow-up data were unavailable for some patients. Furthermore, assessment of neurological function and data collection were primarily based on clinical records, which might be subject to recall bias and lacked standardized, quantitative functional scoring systems. These limitations may have affected the accuracy of correlating radiological changes with functional outcomes. Moreover, there is still inadequate evidence to comprehensively support the hypothesis that multiple segmental syrinxes in patients with VHL-associated spinal hemangioblastomas may gradually merge into a holocord syringomyelia. Therefore, the conclusions derived from this study still require further validation in larger cohorts with long-term, systematic follow-up.

In future, emerging imaging techniques may further refine management. DTI of the spinal cord could help identify the safest site for fenestration by mapping white matter tracts, minimizing iatrogenic injury in patients with thinned cords (27). Radiomics, through high-dimensional feature extraction from routine MRI, may enable prediction of which lesion is most likely to hemorrhage or progress, guiding surveillance and surgical prioritization (28). These approaches remain investigational and require prospective validation in larger cohorts, but represent promising avenues for future research.

## 4 Conclusion

Our study analyzed four VHL-associated holocord syringomyelia cases and, more importantly, provides a novel pathophysiological interpretation and management framework for this rare condition. All patients presented with multiple intramedullary hemangioblastomas and heterogeneous clinical manifestations, uncorrelated with syrinx extent. Its pathogenesis stems from multifocal lesions causing chronic CSF dynamic disorders and abnormal tumor microenvironments, not single tumors. Resecting symptomatic lesions relieved neurological symptoms, but the imaging of syringomyelia may not improve significantly. Thus, an individualized, symptom-focused strategy is advised: follow-up for asymptomatic patients, surgery for symptomatic ones, and radiosurgery/targeted therapy (belzutifan) as adjuvants. Limited by small sample and non-standard assessment, our hypotheses need validation in large, long-term cohort studies.

## Data Availability

The raw data supporting the conclusions of this article will be made available by the authors, without undue reservation.

## References

[1] Mortazavi A Nwokoye D Asuzu DT Scott G Mastorakos P Chittiboina P. Multiple VHL-related hemangioblastomas and holocord syrinx: identifying the causative lesion. Illustrative case. J Neurosurg Case Lessons. (2021) 2:CASE21296. 10.3171/CASE21296 35855304 PMC9265196

[2] Lonser RR Weil RJ Wanebo JE DeVroom HL Oldfield EH. Surgical management of spinal cord hemangioblastomas in patients with von Hippel-Lindau disease. J Neurosurg. (2003) 98:106–16. 10.3171/jns.2003.98.1.0106 12546358

[3] Zheng L Liao Z Duan H. Holocord syringomyelia caused by tethered cord syndrome: case report and literature review. BMC Neurol. (2024) 24:439. 10.1186/s12883-024-03951-2 39528987 PMC11552217

[4] Giner J Pérez López C Hernández B Gómez de la Riva Á Isla A Roda JM. Update on the pathophysiology and management of syringomyelia unrelated to chiari malformation. Neurologia (Engl Ed). (2019) 34:318–25. 10.1016/j.nrl.2016.09.010 27939111

[5] Gardner WJ. Hydrodynamic mechanism of syringomyelia: its relationship to myelocele. J Neurol Neurosurg Psychiatry. (1965) 28:247–59. 10.1136/jnnp.28.3.247 14345682 PMC495899

[6] Kreatsoulas DC Lonser RR. Spinal cord hemangioblastomas in von hippel-lindau disease. Neurooncol Adv. (2024) 6:iii66–72. 10.1093/noajnl/vdad153 39430395 PMC11485647

[7] Yilmaz TF Toprak H Sari L Oz II Kitis S Kaya A. Chiari type 1 malformation: CSF flow dynamics and morphology in the posterior fossa and craniocervical junction and correlation of these findings with syrinx formation. Neurochirurgie. (2022) 68:595–600. 10.1016/j.neuchi.2022.06.001 35752467

[8] Xu L Wu Y Liao Z Shen S Xu F Yi Z. An autologous duraplasty *in situ* technique in the treatment of Chiari malformation type I: a prospective study. Acta Neurol Belg. (2024) 124:1311–7. 10.1007/s13760-024-02579-w 38769273

[9] Lonser RR Vortmeyer AO Butman JA Glasker S Finn MA Ammerman JM. Edema is a precursor to central nervous system peritumoral cyst formation. Ann Neurol. (2005) 58:392–9. 10.1002/ana.20584 16130092

[10] Lonser RR Butman JA Oldfield EH. Pathogenesis of tumor-associated syringomyelia demonstrated by peritumoral contrast material leakage. Case illustration. J Neurosurg Spine. (2006) 4:426. 10.3171/spi.2006.4.5.426 16703915

[11] Wu TC Guo WY Lirng JF Wong TT Chang FC Luo CB. Spinal cord hemangioblastoma with extensive syringomyelia. J Chin Med Assoc. (2005) 68:40–4. 10.1016/S1726-4901(09)70131-5 15742863

[12] Dutta G Singh D Singh H Srivastava AK Jagetia A Agrawal A. Dorsal hemangioblastoma manifesting as holocord syringomyelia. Surg Neurol Int. (2018) 9:73. 10.4103/sni.sni_47_18 29721352 PMC5909086

[13] Borkar SA Kasliwal MK Suri A Sharma BS. Cervical hemangioblastoma with holocord syrinx. Surg Neurol. (2009) 72:437–8. 10.1016/j.surneu.2008.11.014 19329165

[14] Munjal S. Conus hemangioblastoma with holocord syrinx not associated with von-Hippel Lindau (vHL) syndrome: a case report. J Spinal Surg. (2018) 5:144–6. 10.5005/jp-journals-10039-1189

[15] Pai SB Krishna KN. Secondary holocord syringomyelia with spinal hemangioblastoma: a report of two cases. Neurol India. (2003) 51:67–8. 12865521

[16] Chu EC Sabourdy E Cheong B. Secondary holocord syringomyelia associated with spinal hemangioblastoma in a 29-year-old female. Cureus. (2023) 15:e40022. 10.7759/cureus.40022 37287819 PMC10242357

[17] Lee S Chung CK. Microsurgical resection of a large conus medullaris hemangioblastoma associated with holocord syringomyelia: 2-dimensional operative video. Oper Neurosurg. (2023) 25:e295. 10.1227/ons.0000000000000852 37650618

[18] Knoop N Seidel C Frydrychowicz C Meixensberger J. Combined microsurgery and radiotherapy for multiple spinal cord hemangioblastomas with holocord syrinx in von Hippel-Lindau disease: a case report. J Neurol Surg Rep. (2019) 80:e46–50. 10.1055/s-0039-3401808 31908906 PMC6938460

[19] Wanebo JE Lonser RR Glenn GM Oldfield EH. The natural history of hemangioblastomas of the central nervous system in patients with von hippel-lindau disease. J Neurosurg. (2003) 98:82–94. 10.3171/jns.2003.98.1.0082 12546356

[20] Ammerman JM Lonser RR Dambrosia J Butman JA Oldfield EH. Long-term natural history of hemangioblastomas in patients with von Hippel-Lindau disease: implications for treatment. J Neurosurg. (2006) 105:248–55. 10.3171/jns.2006.105.2.248 17219830

[21] Chinese Society of Rare Diseases, Chinese Medical Association; Beijing Society of Rare Diseases; Chinese Urological Association, Chinese Consortium for Genetic and Rare Diseases of the Urinary System. Chinese expert consensus for the diagnosis and treatment of von hippel-lindau syndrome (2025 edition). Zhonghua Yi Xue Za Zhi. (2025) 105:4441–52. (Chinese). 10.3760/cma.j.cn112137-20250624-01538 41456881

[22] Qiu J Cai D Yang F Zhou J Gong Y Cai L. Stereotactic radiosurgery for central nervous system hemangioblastoma in von hippel-lindau disease: a systematic review and meta-analysis. Clin Neurol Neurosurg. (2020) 195:105912. 10.1016/j.clineuro.2020.105912 32474257

[23] Oldfield EH. Editorial: management of hemangioblastomas in patients with von Hippel-Lindau disease: stereotactic radiosurgery compared to surgical excision. J Neurosurg. (2015) 122:1466–8. 10.3171/2015.2.JNS15252 25816089

[24] Courtney KD Ma Y Diaz de Leon A Christie A Xie Z Woolford L. HIF-2 complex dissociation, target inhibition, and acquired resistance with pt2385, a first-in-class hif-2 inhibitor, in patients with clear cell renal cell carcinoma. Clin Cancer Res. (2020) 26:793–803. 10.1158/1078-0432.CCR-19-1459 31727677 PMC7024660

[25] Wach J Basaran AE Vychopen M Tihan T Wostrack M Butenschoen VM. Local tumor control and neurological outcomes after surgery for spinal hemangioblastomas in sporadic and von hippel-lindau disease: a multicenter study. Neuro Oncol. (2025) 27:1567–78. 10.1093/neuonc/noaf041 39950840 PMC12309710

[26] Cosgrove J Warren D Derham C Jamieson S. Holocord syrinx associated with haemangioblastoma. Pract Neurol. (2015) 15:485–7. 10.1136/practneurol-2015-001251 26438882

[27] Talbott JF Shah V Ye AQ. Diffusion imaging of the spinal cord: clinical applications. Neurosurg Clin N Am. (2025) 36:541–53. 10.1016/j.nec.2025.04.010 41167824

[28] Zhang Z Li N Qian Y Cheng H. Establishment of an MRI-based radiomics model for distinguishing between intramedullary spinal cord tumor and tumefactive demyelinating lesion. BMC Med Imaging. (2024) 24:317. 10.1186/s12880-024-01499-8 39574000 PMC11583559

